# Early Cellular Responses Induced by Sedimentary Calcite-Processed Particles in Bright Yellow 2 Tobacco Cultured Cells

**DOI:** 10.3390/ijms21124279

**Published:** 2020-06-16

**Authors:** Daniel Tran, Tingting Zhao, Delphine Arbelet-Bonnin, Takashi Kadono, Patrice Meimoun, Sylvie Cangémi, Camille Noûs, Tomonori Kawano, Rafik Errakhi, François Bouteau

**Affiliations:** 1 Agroscope, Institute for Plant Production Systems, 1964 Conthey, Switzerland; 2 Laboratoire Interdisciplinaire des Energies de Demain, Université de Paris, 75013 Paris, France; zhaotingting0325@gmail.com (T.Z.); delphine.bonnin@univ-paris-diderot.fr (D.A.-B.); kadono.takashi@gmail.com (T.K.); patrice.meimoun@upmc.fr (P.M.); sylvie.cangemi@univ-paris-diderot.fr (S.C.); francois.bouteau@univ-paris-diderot.fr (F.B.); 3 Graduate School of Environmental Engineering, University of Kitakyushu 1-1, Hibikino, Wakamatsu-ku, Kitakyushu 808-0135, Japan; kawanotom@gmail.com; 4 Cogitamus Laboratory, 75013 Paris, France; camille.nous@noussommesluniversite.fr; 5 LINV Kitakyushu Research Center (LINV@Kitakyushu), Kitakyushu 808-0135, Japan; 6 International Photosynthesis Industrialization Research Center, The University of Kitakyushu, Kitakyushu 808-0135, Japan; 7 Paris Interdisciplinary Energy Research Institute (PIERI), Université de Paris, 75013 Paris, France; 8 Eurofins Agriscience Service, Casablanca 20000, Morocco; r.errakhi@gmail.com

**Keywords:** tobacco, calcium, calcite, reactive oxygen species, ion channels, cellular signalization

## Abstract

Calcite processed particles (CaPPs, Megagreen^®^) elaborated from sedimentary limestone rock, and finned by tribomecanic process were found to increase photosynthetic CO_2_ fixation grapevines and stimulate growth of various cultured plants. Due to their processing, the CaPPs present a jagged shape with some invaginations below the micrometer size. We hypothesised that CaPPs could have a nanoparticle (NP)-like effects on plants. Our data show that CaPPs spontaneously induced reactive oxygen species (ROS) in liquid medium. These ROS could in turn induce well-known cellular events such as increase in cytosolic Ca^2+^, biotic ROS generation and activation of anion channels indicating that these CaPPs could activate various signalling pathways in a NP-like manner.

## 1. Introduction

Several minerals have been used in agriculture [1], among which sedimentary rock that emerges from calcareous seaweed. Megagreen^®^ is a preparation from calcite processed particles (CaPPs), elaborated from sedimentary limestone rock, which is finned and activated by a tribomecanic process [2]. These processed calcite particles are supposedly small enough to enter the leaf and have a beneficial effect on plants. The application of CaPPs on grapevines submitted to water stress was shown to increase photosynthetic CO_2_ fixation [3]. The benefits of CaPPs once inside the plant were supposed to be due the decomposition products, CO_2_ and CaO, that could feed the plant. However, the cellular responses induced by the CaPPs are poorly understood. Due to the tribomecanic processing, the CaPPs present jagged shape with some invaginations below the micrometer size (Megagreen^®^ data sheet: https://dokumen.tips/documents/megagreen-study.html, accessed on 06/04/2020). Nanoparticles (NPs) possess a large specific surface area allowing a greater reactivity compared to macrosized particles. Since the high surface reactivity of NPs is important for their biological effects, we hypothesised that CaPPs could have NP-like effects on plants.

Recent reviews focused on beneficial applications of nanomaterials in agricultural production [4,5,6,7,8]. NPs notably could induce enhancement in growth and seed yield [9], and participate in crop protection [4,10]. Although some cerium oxide nanoparticles were shown to augment reactive oxygen species (ROS) scavenging in *Arabidopsis thaliana* plants [11], a part of the biological effects of various NPs is proposed to be due to their ability to produce ROS, possibly due to molecular size, shape, oxidation status, increased specific surface area, bonded surface species, surface coating, solubility, and degree of aggregation and agglomeration [12,13,14]. We effectively showed by using *Nicotiana tabacum* L. cv. Bright Yellow 2 (BY-2) cultured cells that TiO_2_ NPs spontaneously generate ROS in the culture medium, but also induced a rapid biological ROS production and a ROS-dependent increase in cytosolic calcium ([Ca^2+^]_cyt_) [15]. Variations of [Ca^2+^]_cyt_ serve as secondary messenger involved in many adaptation and developmental processes in plants [16,17]. Reactive oxygen species also play a key signal transduction role in plant cells, such as growth regulation, development, responses to environmental stimuli and cell death [18,19]. However, the response of plants to NPs varies with the growth stages, type of plant species and the nature of NPs. Thus, they could have positive and negative effects on plants [20]. In this study, we tested the impact of CaPPs on cell viability and further checked if CaPPs as NPs could induce ROS generation due to their increased specific surface area and carried out an experimental layout on plant cultured cells to study the impact of CaPPs on variations of [Ca^2+^]_cyt_, biological ROS generation and ion fluxes variations, early cellular responses frequently involved in signalling processes [21]. 

## 2. Results

### 2.1. Non-Biological ROS Production by CaPPs 

**Figure 1:**
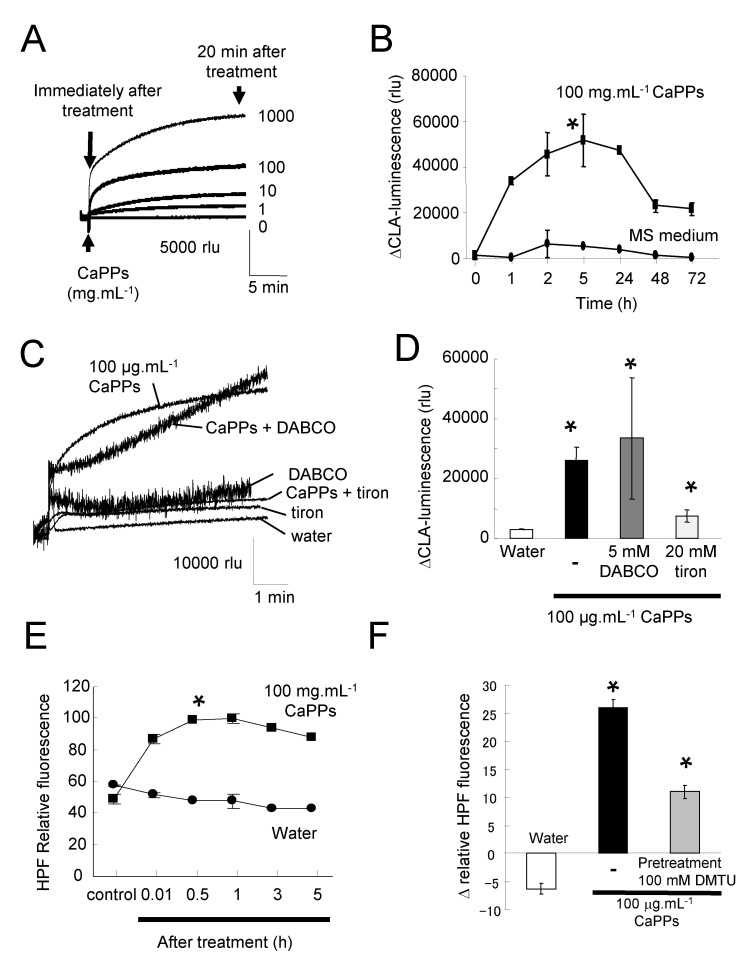
Calcite processed particles (CaPPs)-induced ROS generation in free Murashige and Skoog (MS) medium. **(A**). Typical time and dose *Cypridina* Luminescent Analog (CLA) luminescence recorded in MS medium free of cells after addition of CaPPs. (**B**). Mean values of CaPP-induced CLA luminescence. (**C**,**D**). Effect of singlet oxygen scavenger DABCO (5 mM), and superoxide anion scavenger tiron (20 mM) on CaPP-induced CLA luminescence. The histogram represents the mean values after 20 min. (**E**). Time-dependent hydroxyphenyl fluorescein (HPF) fluorescence in response to 100 µg.mL^−1^ CaPPs. (**F**). Effect of hydroxyl radical scavenger DMTU (100 mM) on CaPP-induced HPF fluorescence after 30 min. Data corresponded to mean values ± standard error (SE) of at least 4 independent experiments. * Significantly different from the water treatment. Data were analyzed by variance analysis (ANOVA) and when ANOVA gave a statistically significant result, the Newman–Keuls multiple range test was used to identify which specific pairs of means were different. All numeric differences in the data were considered significantly different for a *p*-value ≤ 0.05.

We made the hypothesis that CaPPs could have a NP-like effects and could thus generate ROS independently of living cells. According to this hypothesis, we checked if CaPPs could induce ROS generation independently of any living cells. We showed by using the Murashige and Skoog (MS) culture medium that CaPPs spontaneously generate in a dose- and time-dependent manner ROS production evidenced by chemiluminescence of *Cypridina* luciferin analogue (CLA) (Figure 1A,B, Supplemenary Figure S1A). It is noteworthy that, on the contrary to CaPPs, the addition of dissolved CaCO_3_ (the main component of CaPPs) at 100 µg.mL^−1^ in free MS medium did not induce ROS generation (Supplementary Figure S1B), reducing the likelihood of a chemical effect for CaPPs and providing a NP-like effect. The CaPP-induced ROS production continues to increase for about 5 h and decreases slowly after 24 h (Figure 1B). The chemiluminescence of CLA indicates the generation of superoxide anion (O_2_^•−^), and of singlet oxygen (^1^O_2_) to a lesser extent [22]. We then checked the effect of DABCO (1,4-diazabicyclo(2,2,2)octane, a scavenger of ^1^O_2_) and tiron (sodium 4,5-dihydroxybenzene-1,3-disulfonate, a scavenger of O_2_^•−^) on CaPP-induced ROS generation (Figure 1C,D). Only tiron allowed for a significant decrease of ROS generation. This suggests that CaPPs induced mainly O_2_^•−^ generation in culture medium. Since hydroxyl radical (HO^•^) could be chemically generated from O_2_^•−^ through Haber–Weiss or Fenton reactions, we further search for HO^•^ generation by using the specific probe hydroxyphenyl fluorescein (HPF) [23]. A time-dependent increase in HPF fluorescence could be detected upon treatment with 100 µg.mL^−1^ CaPPs (Figure 1E). This increase in HPF fluorescence was decreased by a pretreatment with 100 mM DMTU (Dimethylthiourea), a scavenger of HO^•^ (Figure 1F) supporting the hypothesis of HO^•^ generation. 

### 2.2. CaPP Particles Induced Cytosolic Calcium Variation in Tobacco BY-2 Cells 

**Figure 2:**
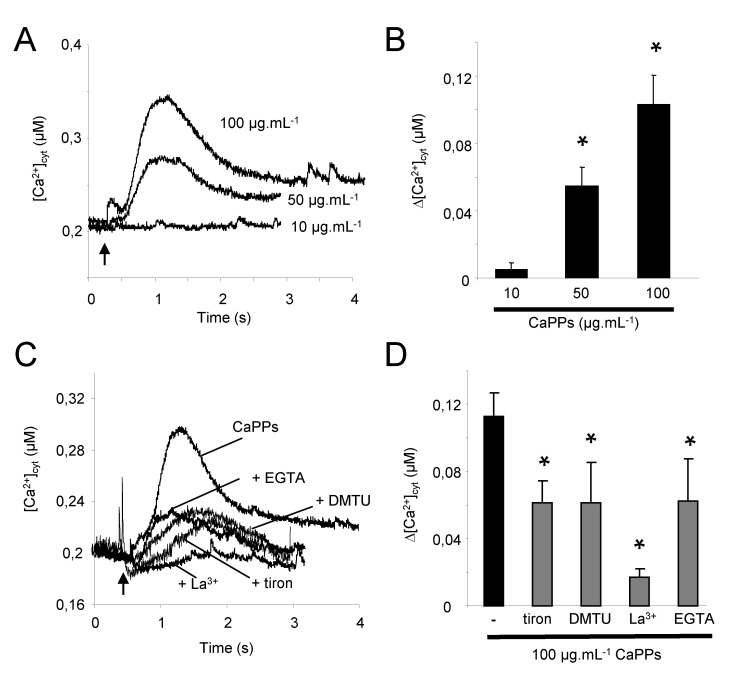
CaPP-induced variations of cytosolic Ca^2+^ in BY-2 cells. (**A**). A typical [Ca^2+^]_cyt_ variations of aequorin expressing *BY-2* cells in response to various concentrations of CaPPs. (**B**). Mean values of maximal [Ca^2+^]_cyt_ increase in response to various concentrations of CaPPs. * Significantly different from the treatment at 10 µg.mL^−1^ CaPPs. (**C**). Effect of calcium (La^3+^, EGTA) and ROS (tiron and DMTU) pharmacology on 100 mg.ml^−1^ CaPPs induced [Ca^2+^]_cyt_ variations. (**D**). Mean values of maximal [Ca^2+^]_cyt_ increase in response to 100 µg.mL^−1^ of CaPPs in the presence of calcium and ROS pharmacology. Controls with pharmacology alone did not affect significantly the basal [Ca^2+^]_cyt_ (not shown). Data corresponded to mean values ± SD of at least six independent experiments. * Significantly different from the treatment at 100 µg.mL^−1^. Data were analyzed by variance analysis (ANOVA) and when ANOVA gave a statistically significant result, the Newman–Keuls multiple range test was used to identify which specific pairs of means were different. All numeric differences in the data were considered significantly different for a *p*-value ≤ 0.05.

We showed that TiO_2_ NPs induced a ROS-dependent increase in cytosolic calcium ([Ca^2+^]_cyt_) in BY-2 tobacco cells [15]. ROS were also shown to activate plasma membrane Ca^2+^ channels in plant cells [24]. We thus investigated the effect of CaPPs on cytosolic calcium level in BY-2 tobacco cultured cells expressing the Ca^2+^-sensitive luminescent protein aequorin in their cytosol [25]. CaPPs induced a rapid dose-dependent and transient increase in [Ca^2+^]_cyt_ (Figure 2A,B). Influx of Ca^2+^ from the apoplast through plasma membrane was confirmed by using 500 µM La^3+^, a blocker of Ca^2+^ channels, and 3 mM EGTA, a calcium chelator (Figure 2C,D). This Ca^2+^ influx was dependent on the early CaPP-induced ROS production since tiron, and DMTU could also reduce the [Ca^2+^]_cyt_ increase (Figure 2C,D). 

Variations in [Ca^2+^]_cyt_ and ROS generation are known to regulate different early events involved in signal transduction pathways such as ion channel activities and NADPH-oxidase activities induced in response to various biotic and abiotic stressors [21,26]. We then further checked if such events could be regulated by CaPPs.

### 2.3. CaPPs Induced a NADPH Oxidase-Dependent ROS Production 

**Figure 3:**
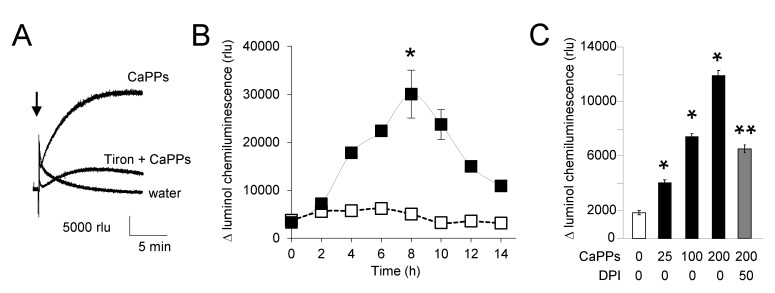
Biological CaPP-induced ROS generation by BY-2 cells. (**A**). Typical time CLA luminescence recorded with BY-2 cells after addition of 100 µg.mL^−1^ CaPPs with or without 20 mM tiron. (**B**). Kinetic of biological ROS generation detected with luminol during 14 h after addition of 100 µg.mL^−1^ CaPPs. (**C**). Mean values of maximal ROS increase (at 8h) in response to various CaPPs concentrations (in mg.mL^−1^) and in the presence 50 μM diphenyleneiodonium (DPI), an inhibitor of NADPH-oxidase. Data corresponded to mean values ± SD of at least six independent experiments. * significantly different from the control. ** Significantly different from the treatment at 200 µg.mL^−1^ CaPPs. Data were analyzed by variance analysis (ANOVA) and when ANOVA gave a statistically significant result, the Newman–Keuls multiple range test was used to identify which specific pairs of means were different. All numeric differences in the data were considered significantly different for a *p*-value ≤ 0.05.

As expected from the spontaneously CaPP-induced ROS production in MS medium (Figure 1A), the chemiluminescence of CLA also rapidly increased after addition of 100 µg.mL^−1^ CaPPs in BY-2 cell cultures (Figure 3A). From analysis of luminol-chemilumiscence, we further showed that CaPP-induced ROS generation reached a maximum at about 8 h in BY-2 cultured cells when untreated cells presented no significant increase in chemilumiscence level during the time of experiments (Figure 3B). This effect was dose-dependent (Figure 3C). The addition of 50 μM diphenyleneiodonium (DPI), an inhibitor of NADPH-oxidase [27,28], into BY-2 cell medium diminished the chemilumiscence (Figure 3C). These data suggest the involvement of plant enzymes such NADPH-oxidase in this ROS production induced by CaPPs. 

### 2.4. CaPPs Induce a Depolarization of Plasma Membrane Due to Anion Channel Activation 

**Figure 4:**
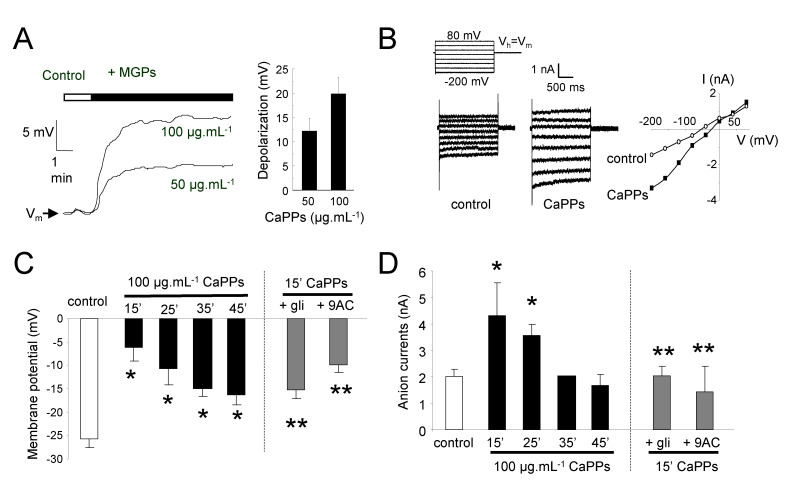
CaPP-induced depolarization and anion current increase in BY-2 cells. (**A**). Typical depolarizations of BY-2 cell observed in response to CaPPs at 50 or 100 µg.mL^−1^ and mean values of depolarizations. (**B**). Whole currents measured under control conditions and 5 min after addition of 100 µg.mL^−1^ CaPPs. The protocol was as illustrated, holding potential (V_h_) was V_m_. Corresponding current-voltage relationships at 1.8 s. (**C**). Mean values of polarizations for BY-2 cells treated during different times with 100 µg.mL^−1^ CaPPs and mean values of polarizations for BY-2 cells treated 15 min with 100 µg.mL^−1^ CaPPs in the presence of 200 µM glibenclamide (gli) or 200 µM 9-antharcen carboxylic acid (9AC), two unrelated anion channel inhibitors. (**D**). Mean values of anion currents for BY-2 cells treated during different times with 100 µg.mL^−1^ CaPPs and mean values of anion currents for BY-2 cells treated 15 min with 100 µg.mL^−1^ CaPPs in the presence of 200 µM gli or 200 µM 9AC. Currents were recorded at −200 mV and 1.8 s of voltage clamp. Control values corresponded to the value before CaPPs addition. Data corresponded to mean values ± SD of at least six independent experiments. * Significantly different from the control. ** Significantly different from the treatment at 15 min. Data were analyzed by variance analysis (ANOVA) and when ANOVA gave a statistically significant result, the Newman–Keuls multiple range test was used to identify which specific pairs of means were different. All numeric differences in the data were considered significantly different for a *p*-value ≤ 0.05.

We used an electrophysiological approach to test the effect of CaPPs on membrane potentials and ion currents of cultured cells. Upon direct addition of CaPPs, we recorded a rapid dose-dependent depolarization of BY-2 cells (Figure 4A). The depolarization was correlated with a large increase in ion currents (Figure 4B). Because impalement of a single cells could not be maintained for a long time, we further analysed the mean plasma membrane potentials and ion currents of BY-2 cell populations exposed to CaPPs for different amounts of time (Figure 4C,D). The value of the resting membrane potential (V_m_) of control cells (without treatment) was around -25 mV (Figure 4C), in the same range of previous studies [26,29]. As expected from the direct addition of CaPPs (Figure 4A), cells pretreated 15 min with CaPPs were drastically depolarized (Figure 4C), but these depolarizations were transient and the cell polarizations were partly recovered for cells pretreated during 45 min (Figure 4C). These membrane potential variations were correlated with a transient increase in ion currents (Figure 4B,D) presenting the main hallmarks of anion current as previously characterized [26,29,30,31]. This type of current was shown to be sensitive to structurally unrelated anion channel inhibitors [26,29]. Accordingly, the increases in ion currents and the depolarizations after 15 min CaPPs pretreatment were effectively partly avoided upon pretreatment with 200 µM of glibenclamide (gli) or 9-anthracen carboxylic acid (9AC), two structurally unrelated anion channel blockers (Figure 4D), confirming the anionic nature of these currents. These currents present the features of slow anion channels [32], but a part of the instantaneous current could be carried out by fast-activating anion channels [33]. However, these data show that increase in anion currents could be part of the early CaPP-induced signaling events.

### 2.5. CaPPs Toxicity?

**Figure 5:**
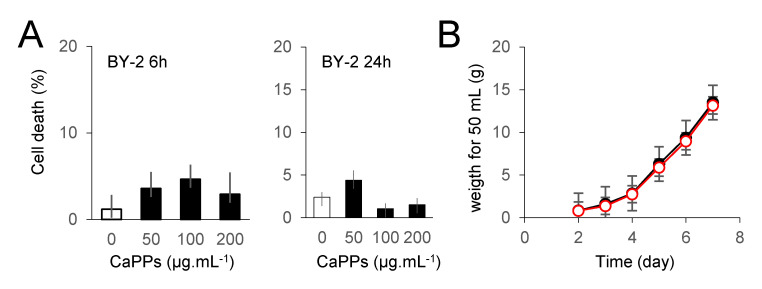
CaPPs cytotoxicity in *BY-2* cultured cells. (**A**). Cell death extent in BY-2 cultured cells detected by the Evans Blue staining after 6 or 24h of treatment with various concentrations of CaPPs. (**B**). BY-2 cultured cell growth during 7 days in the presence or not of 200 µg.mL^−1^ CaPPs. The data corresponded to means of at least 4 independent replicates and error bars corresponded to SE.

Nanoparticles were shown to induce cell death in various models [6,13,34]. We thus checked if CaPPs could induce death of BY-2 cells. No increase in cell death was observed in BY-2 cultured cells, even after 24 h of treatment (Figure 5A). We further checked if these CaPPs could have an impact on BY-2 cell culture growth. As expected from the data of cell death, addition of CaPPs in the culture medium of BY-2 cells for 7 days has no impact on the culture cell growth (Figure 5B).

## 3. Discussion

The CaPPs application has been shown to be beneficial on several crops such as olive trees, maize, strawberry and lettuce, especially under drought conditions (technical data sheet for Megagreen^®^: https://dokumen.tips/documents/megagreen-study.html, accessed on 06/04/2020). The benefits of CaPPs once inside the leaves was attributed to the decomposition products CO_2_ and CaO that could feed the plant. CaPPs application on grapevines subjected to water stress was shown to increase photosynthetic CO_2_ fixation [3]. The CaPPs penetrating directly into the leaves are supposed to increase CO_2_ saturation in the leave leading to stomatal closure and therefore a reduction in evapotranspiration a reduction of photorespiration in favor of photosynthesis [3]. Spray of CaO were also shown to correct Ca^2+^ deficiency in groundnut [35] although the mean levels of Ca^2+^ were not statistically different between CaPP-treated and untreated vines [36]. However, due to the size distribution of these CaPPs ranging from the nano- to the microparticle (0.1 to 20 μm), we hypothesized NP-like effects of CaPPs at the cellular level. By using nonphotosynthetic BY-2 cultured plant cells, we could discriminate the effects of NPs from already-reported effects on photosynthetic activity. 

Our data showed that CaPPs induce ROS generation independently of any living cells. This ROS production is dose- and time-dependent and seemed to be mainly due to O_2_^•−^ (detected by CLA and scavenged by tiron) and subsequently HO^•^ (detected by HPF and scavenged by DMTU) through Haber–Weiss or Fenton reactions after the dismutation of O_2_^•−^ into H_2_O_2_. These data correlate with previous one indicating that NPs from different nature can produce ROS due to their increased specific surface area [12,13,14]. 

Our pharmacological data with ROS scavengers show that these CaPP-induced ROS could be responsible in BY-2 cells for the induction of well-known cellular events involved in the signalling process, such as calcium influx through plasma membrane Ca^2+^ channels, subsequent NADPH-oxidase stimulation and anion channel activation. The NADPH-oxidase stimulation and anion channel activations could also be recorded in response to CaPPs in *A. thaliana* cultured cells (Supplementary Figure S2). ROS generation and the cytosolic calcium increase are reminiscent with what was observed in response to TiO_2_ NPs in BY-2 cells [15], or in response to ZnO NPs in *Salicornia* [37], but also in responses to O_3_, another oxidative stress [38], on tobacco cells [39] and *A. thaliana* cultured cells [21,40,41,42]. Less data are available on the effect of NPs on ion channel regulation especially in plants, but it is noticeable that polystyrene NPs could activate CFTR-Cl^−^ channels in hamster kidney cells [43] and O_3_ anion currents in *A. thaliana* cells [21]. 

Although CaPPs do not seem to be toxic for BY-2 cells, such signalling events are frequently related to the induction of programmed cell death (PCD) [21,26,29]. Effectively we could observe in *A. thaliana* cells after addition of CaPPs an increase in cell death slowing the whole suspension growth (Supplementary Figure S3). Toxic effects of nanoparticles were already observed in response to various NPs such as ZnONPs or AgNPs in algae [44,45] or CuONPs, SiNPs and single-wall carbon nanotubes on a terrestrial model [34,46,47], sometimes due to the PCD process [34]. In *A. thaliana,* cell death was dependent on transcription and translation (Figure S3), effectively suggesting an active process, thus a PCD. The discrepancy observed in terms of cell death between the two cultured cell lines, since there was no record of cell death or the slowing of suspension cell growth for BY-2 cells, which could be explained by a difference in sensitivity. Effectively, carbon nanotubes were shown to induce the growth enhancement of tobacco cells [48] when they induce PCD in *A. thaliana* and rice [14,34]. However, the CaPP-induced PCD in *A. thaliana* cells could be reduced by the ROS scavengers DMTU and tiron, the blockers of Ca^2+^ influx, BAPTA and La^3+^ and the anion channel blockers 9AC and glibenclamide (Supplementary Figure S3). These data support the hypothesis that the CaPP-induced ROS generation induces the signaling pathways leading to the PCD process. It is also noteworthy that these cellular events are also involved in stomatal aperture regulation [49]. We could further confirm the decrease of stomatal aperture 30 min after application of 50 μM CaPPs on the epidermis *A. thaliana* leaves (Supplementary Figure S4). Thus, the CaPP-induced stomatal closure could be due to not only an increase in CO_2_ saturation of the leaves [3], but also to the CaPP-induced ROS generation. 

In summary, our study shows that CaPPs could have, in addition to its known effects on photosynthesis [3], NP-like effects due to their size distribution. The abiotic ROS generation induced by these CaPPs could induce cellular events that could be involved in various signaling pathways. More studies, particularly with different species, will be needed to clarify the possible outputs of these signaling pathways. 

## 4. Materials and Methods

### 4.1. CaPP Particles 

Megagreen^®^ is composed of calcite processed particles (CaPPs) elaborated from sedimentary limestones rock, which is finned and activated by tribomecanic process (European Patent N° WO/2000/064586). These CaPPs present a distribution ranging from the nano- to the microparticle (0.1 to 20 μm). The chemical composition of CaPPs is: total calcium carbonate 823.0 g∙kg^−1^; SiO_2_ 85.2 g kg^−1^; MgO 30.2 g∙kg^−1^; Fe 8.78 g∙kg^−1^, and other trace elements. CaPPs were diluted in distilled water and pH adjusted to 5.8 with HCl. 

### 4.2. Plant Cell Culture Conditions 

*Nicotiana tabacum* BY-2 cultured cells were grown in Murashige and Skoog medium (MS medium) [50] complemented with 30 g.L^−1^ sucrose, 0.1 mg.L^−1^ 2,4 D (pH 5.8) and maintained by weekly dilution (2/80). The cell culture was agitated on a rotary shaker at 120 rpm at 22 ± 2 °C in the dark. Such cells are white and nonphotosynthetic. All experiments were performed at 22 ± 2 °C using the cells in log-phase (6 days after subculturing). 

Cell growth was estimated for by recording each day after subculture the fresh weight of cells contained in 50 mL of culture for BY-2 cell cultures. 

### 4.3. Monitoring of ROS Production

The production of ROS was monitored using different techniques and probes. The chemiluminescence of the *Cypridina* luciferin analog (CLA) react mainly with O_2_^•−^ and ^1^O_2_ with light emission [22]. Chemiluminescence from CLA was monitored using a FB12-Berthold luminometer (with a signal integrating time of 0.2 s). For data analysis, the luminescence ratio (L/Lbasal) was calculated by dividing the luminescence intensities of CLA-luminescence (L) with the luminescence intensity before treatment (Lbasal). Hydroxy radicals (HO^•^) formation was also checked using the specific probe hydroxyphenyl fluorescein (HPF) [23]. Briefly, HPF was added to 1mL of MS medium to a final concentration of 10 µM at different times after the addition of 100 mg.mL^−1^ of CaPPs. The fluorescence increase was monitored at 515 nm after an excitation at 490 nm using a F-2000 spectrofluorimeter (Hitachi, Tokyo, Japan). 

For biological production of ROS, we used the chemiluminescence of luminol [51], which is dependent on the activity of cell-derived peroxidase. Briefly, 6 mL of the cultured cells were inoculated with CaPPs. Before each measurement, 200 µL of the cell culture was added prior to the addition of 5 µL luminol (1.1 mM). Chemiluminescence measurements were carried out at 30 min intervals using a FB12-Berthold luminometer (signal integrating time 0.2 s). 

### 4.4. Aequorin Luminescence Measurements 

Cytoplasmic Ca^2+^ variations were recorded from BY-2 cultured cells expressing the apoaequorin gene [25]. For Ca^2+^ measurement, aequorin was reconstituted by an overnight incubation of the cell cultures in MS medium supplemented with 2.5 µM native coelenterazine. Cell culture aliquots (450 µL in MS medium) were transferred carefully to a luminometer glass tube and luminescence was recorded continuously at 0.2 s intervals using a FB12-Berthold luminometer (Berthold Technologies, Bad Wildbad, Germany). Treatments were performed by 50 µL injections containing the CaPPs. At the end of each experiment, residual aequorin was discharged by addition of 500 µL of a 1M CaCl_2_ solution dissolved in 100% methanol. The resulting luminescence was used to estimate the total amount of aequorin in each experiment. Calibration of the calcium measurement was performed using the equation: pCa = 0.332588(−logk) +5.5593, where k is a rate constant equal to luminescence counts per second divided by total remaining counts [25]. To test the effects of each different pharmacological treatment, BY-2 cells were pretreated for 15 min before the application of CaPPs. 

### 4.5. Electrophysiology

Experiments were conducted on BY-2 cells maintained in their culture medium to limit stress (main ions in MS medium 28 mM NO_3_^−^ and 16 mM K^+^) [26]. Individual cells were immobilized by a microfunnel (approximately 30 to 80 µm outer diameter and controlled by a micromanipulator (WR6-1, Narishige, Tokyo, Japan). Impalement were carried out with a piezoelectric micromanipulator (PCS-5000, Burleigh Inst., New York, NY, USA) in a chamber (500 µL) made of Perspex. Voltage-clamp measurements of whole-cell currents from intact BY-2 cells presenting stable running membrane potential were carried out at room temperature (20–22 °C) using the technique of the discontinuous single voltage-clamp microelectrode [52] adapted to plant cells [40,53]. Microelectrodes were made from borosilicate capillary glass (Clark GC 150F, Clark Electromedical, Pangbourne Reading, UK) pulled on a vertical puller (Narishige PEII, Tokyo, Japan). Their tips were less than 1 µm diameter; they were filled with 600 mM KCl, and had electrical resistances between 20 and 50 MΩ with the culture medium. Specific software (pCLAMP 8) drives the voltage clamp amplifier (Axoclamp 2A, Molecular Devices, Sunnyvale, CAL, USA). Voltage and current were digitalised with a Digidata 1322A (Molecular Devices, Sunnyvale, CAL, USA). In whole-cell current measurements the membrane potential was held to the value of the resting membrane potential. Current recordings were obtained by hyperpolarizing pulses from −200 to +80 mV (20 mV, 2 s steps of current injection, 6s of settling time). We systematically checked that cells were correctly clamped by comparing the protocol voltage values with those really imposed. Only microelectrodes presenting a linear relationship were used. 

### 4.6. Cell Viability Assays

Cell viability was checked using the vital dye, Evans Blue. Cells (50 μL) were incubated for 5 min in 1 mL phosphate buffer pH 7 supplemented with Evans blue to a final concentration of 0.005%.^21^ Cells that accumulated Evans blue were considered dead. At least 1000 cells were counted for each independent treatment. The experiment was repeated at least 4 times for each condition.

### 4.7. Statistical Analysis 

Data were analyzed by variance analysis (ANOVA) and when ANOVA gave a statistically significant result, the Newman–Keuls multiple range test was used to identify which specific pairs of means were different. All numeric differences in the data were considered significantly different for a *p*-value ≤ 0.05.

## Supplementary Materials

Supplementary Materials can be found at https://www.mdpi.com/1422-0067/21/12/4279/s1. Figure S1. A. Mean values of CaPP-induced CLA luminescence B. Comparison of CaPP- and CaCO_3_-induced ROS generation in free MS medium. Figure S2: **A.** Kinetic of biological ROS generation detected with luminol during 7 h after addition of 100 µg.mL^−1^ CaPPs. **B.** Mean values of polarizations for A. thaliana cells treated during different times with 100 µg.mL^−1^ CaPPs and mean values of polarizations for A. thaliana cells treated 15 min with 100 µg.mL^−1^ CaPPs in presence of 200 µM glibenclamide (gli) or 200 µM 9-antharcen carboxylic acid (9AC), two unrelated anion channel inhibitors. Figure S3: A. Dose-dependent cell death reaching about 50% of the *Arabidopsis thaliana* cell population was observed after 6 h after treatment with 200 µg. mL^−1^ CaPPs. B. Decrease of the culture growth induced by 200 µg. mL^−1^ CaPPs. C. Decrease of cell death extent by pretreatments with actinomycin D (AD, 20 μg/mL), cycloheximide (Chx, 20 μg/mL), inhibitors of traduction and translation, ROS scavengers Tiron (5 mM) and DMTU (100 mM), Ca^2+^ channel blocker La^3+^ (500 µM), Ca^2+^ chelator, BAPTA (3 mM), and anion channel blockers, glibenclamide (gli 200 µM) and 9AC (200 µM). For each pretreatment, cells were incubated for 15 min before CaPPs treatment. Figure S4: Applications of 100 µg mL^−1^ CaPPs reduce the stomatal aperture of *A. thaliana* leaves. Figure S4: Applications of 100 µg.mL^−1^ CaPPs reduce the stomatal aperture of *A. thaliana* leaves. In presence of 3 mM EGTA, the CaPPs-induced stomatal closure was reduced.

## References

[1] Van Straaten P. (2006). Farming with Rocks and Minerals: Challenges and Opportunities. An. Acad. Bras. Ciênc..

[2] (2000). Device for Micronizing Materials. EU Patent.

[3] Attia F., Martinez L., Lamaze T. (2014). Foliar Application of Processed Calcite Particles Improved Leaf Photosynthsis of Potted *Vitis vinifera* Grown Under Water Deficit. OENO ONE.

[4] Khot L.R., Sankaran S., Maja J.M., Eshani R., Schuster E.W. (2012). Applications of Nanomaterials in Agricultural Production and Crop Protection. Crop Prot..

[5] Parisi C., Vigani M., Rodríguez-Cerezo E. (2015). Agricultural Nanotechnologies: What Are the Current Possibilities?. Nano Today.

[6] Wang P., Lombi E., Zhao F.J., Kopittke P.M. (2016). Nanotechnology: A New Opportunity in Plant Sciences. Trends Plant Sci..

[7] Tripathi D.K., Singh S., Singh S., Pandey R., Singh V.P., Sharma N.C., Prasad S.M., Dubey N.K., Chauhan D.K. (2017). An Overview on Manufactured Nanoparticles in Plants: Uptake, Translocation, Accumulation and Phytotoxicity. Plant Physiol. Biochem..

[8] Iavicoli I., Leso V., Beezhold D.H., Shvedova A.A. (2017). Nanotechnology in Agriculture: Opportunities, Toxicological Implications, and Occupational risks. Toxicol. Appli. Pharmacol..

[9] Arora S., Sharma P., Kumar S., Nayan R., Khanna P.K., Zaidi M.G.H. (2012). Gold-Nanoparticles Induced Enhancement in Growth and Seed Yield of *Brassica juncea*. Plant Growth Regul..

[10] Rai M., Ingle A. (2012). Role of Nanotechnology in Agriculture with Special Reference to Management of Insect Pests. Appl. Microbiol. Biotechnol..

[11] Wu H., Tito N., Juan P., Giraldo J.P. (2017). Anionic Cerium Oxide Nanoparticles Protect Plant Photosynthesis from Abiotic Stress by Scavenging Reactive Oxygen Species. ACS Nano.

[12] Oberdöster G., Oberdöster E., Oberdöster J. (2005). Nanotoxicology: An Emerging Discipline Evolving from Studies of Ultrafine Particles. Environ. Health Respect.

[13] Voinov M.A., Sosa J.A., Morrison P.E., Smirnova T.I., Smirnov A.I. (2011). Surface-Mediated Production of Hydroxyl Radicals as a Mechanism of Iron Oxide Nanoparticle Biotoxicity. J. Am. Chem. Soc..

[14] Fu P.P., Xia Q., Hwang H.M., Ray C.P., Yu H. (2014). Mechanisms of Nanotoxicity: Generation of Reactive Oxygen Species. J. Food Drug Anal..

[15] Tran D., Kadono T., Meimoun P., Kawano T., Bouteau F. (2010). TiO_2_ Nanoparticles Induce ROS Generation and Cytosolic Ca^2+^ Increases on BY-2 Tobacco Cells: A Chemiluminescence Study. Luminescence.

[16] Lecourieux D., Ranjeva R., Pugin A. (2006). Calcium in Plant Defence-Signalling Pathways. New Phytol..

[17] Batistič O., Kudla J. (2012). Analysis of Calcium Signaling Pathways in Plants. Biochim. Biophys. Acta.

[18] Foyer C.H., Noctor G. (2009). Redox Regulation in Photosynthetic Organisms: Signaling, Acclimation, and Practical Implications. Antioxid. Redox Signal..

[19] Suzuki N., Miller G., Morales J., Shulaev V., Torres M.A., Mittler R. (2011). Respiratory Burst Oxidases: The Engines of ROS Signaling. Curr. Opin. Plant Biol..

[20] Liu Q., Zhao Y., Wan Y., Zheng J., Zhang X., Wang C., Fang X., Lin J. (2011). Study of the Inhibitory Effect of Water-Soluble Fullerenes on Plant Growth at the Cellular Level. ACS Nano.

[21] Kadono T., Tran D., Errakhi R., Hiramatsu T., Meimoun P., Briand J., Iwaya-Inoue M., Kawano T., Bouteau F. (2010). Increased Anion Channel Activity Is an Unavoidable Event in Ozone-Induced Programmed Cell Death. PLoS ONE.

[22] Nakano M., Sugioka K., Ushijima Y., Goto T. (1986). Chemiluminescence Probe with *Cypridina* Luciferin Analog, 2-methyl-6-phenyl-3,7-dihydroimidazo[1,2-*a*]pyrazin-3-one, for Estimating the Ability of Human Granulocytes to Generate O_2_^−^. Anal. Biochem..

[23] Setsukinai K., Urano Y., Kakinuma K., Majima H.J., Nagano T. (2003). Development of Novel Fluorescence Probes That Can Reliably Detect Reactive Oxygen Species and Distinguish Specific Species. J. Biol. Chem..

[24] Foreman J., Demidchik V., Bothwell J.H., Mylona P., Miedema H., Torres M.A., Linstead P., Costa S., Brownlee C., Jones J.D. (2003). Reactive Oxygen Species Produced by NADPH Oxidase Regulate Plant Cell Growth. Nature.

[25] Knight M.R., Campbell A.K., Smith S.M., Trewavas A.J. (1991). Transgenic Plant Aequorin Reports the Effects of Touch and Cold-Shock and Elicitors on Cytoplasmic Calcium. Nature.

[26] Monetti E., Kadono T., Tran D., Azzarello E., Arbelet-Bonnin D., Biligui B., Briand J., Kawano T., Mancuso S., Bouteau F. (2014). Deciphering in Early Events Involved in Hyperosmotic Stress-Induced Programmed Cell Death in Tobacco BY-2 Cells. J. Exp. Bot..

[27] Murphy T.M., Auh C.K. (1996). The Superoxide Synthases of Plasma Membrane Preparations from Cultured Rose Cells. Plant Physiol..

[28] Van Gestelen P., Asard H., Caubergs R.J. (1997). Solubilization and Separation of a Plant Plasma Membrane NADPH–O_2_- Synthase from Other NADPH Oxidoreductases. Plant Physiol..

[29] Gauthier A., Lamotte O., Reboutier D., Bouteau F., Pugin A., Wendehenne D. (2007). Cryptogein-Induced Anion Effluxes: Electrophysiological Properties and Analysis of the Mechanisms Through Which They Contribute to the Elicitor-Triggered Cell Death. Plant Signal Behav..

[30] Brault M., Amiar Z., Pennarun A.M., Monestiez M., Zhang Z., Cornel D., Dellis O., Knight H., Bouteau F., Rona J.P. (2004). Plasma Membrane Depolarization Induced by Abscisic Acid in Arabidopsis Suspension Cells Involves Reduction of Proton Pumping in Addition to Anion Channel Activation, Which Are Both Ca^2+^ Dependent. Plant Physiol..

[31] Reboutier D., Bianchi M., Brault M., Roux C., Dauphin A., Rona J.P., Legue V., Lapeyrie F., Bouteau F. (2002). The Indolic Compound Hypaphorine Produced by Ectomycorrhizal Fungus Interferes with Auxin Action and Evokes Early Responses in Non-Host *Arabidopsis Thaliana*. Mol. Plant Microbe Interac..

[32] Schroeder J.I., Keller B.U. (1992). Two Types of Anion Channel Currents in Guard Cells with Distinct Voltage Regulation. Proc. Natl. Acad. Sci. USA.

[33] Hedrich R., Busch H., Raschke K. (1990). Ca^2+^ and Nucleotide Dependent Regulation of Voltage Dependent Anion Channels in the Plasma Membrane of Guard Cells. EMBO J..

[34] Shen C.X., Zhang Q.F., Li J., Bi F.C., Yao N. (2010). Induction of Programmed Cell Death in Arabidopsis and Rice by Single-Wall Carbon Nanotubes. Am. J. Bot..

[35] Deepa M., Sudhakar P., Venkata K., Kota N., Reddy B., Krishna T.G., Krishna N.V., Prasad V. (2015). First Evidence on Phloem Transport of Nanoscale Calcium Oxide in Groundnut Using Solution Culture Technique. Appl Nanosci..

[36] Sofo A., Scopa A., Manfra M., De Nisco M., Tenore G.C., Nuzzo V. (2013). Different Water and Light Regimes Affect Ionome Composition in Grapevine (*Vitis Vinifera* L.). Vitis.

[37] Balážová Ľ., Babula P., Baláž M., Bačkorová M., Bujňáková Z., Briančin J., Kurmanbayeva A., Sagi M. (2018). Zinc Oxide Nanoparticles Phytotoxicity on Halophyte from Genus *Salicornia*. Plant Physiol Biochem..

[38] Vainonen J.P., Kangasjärvi J. (2015). Plant Signalling in Acute Ozone Exposure. Plant Cell Environ..

[39] Kadono T., Yamaguchi Y., Furuichi T., Hirono M., Garrec J.P. (2006). Ozone-Induced Cell Death Mediated with Oxidative and Calcium Signaling Pathways in Tobacco Bel-w3 and Bel-B Cell Suspension Cultures. Plant Signal. Behav..

[40] Tran D., El-Maarouf-Bouteau H., Rossi M., Biligui B., Briand J., Kawano T., Mancuso S., Bouteau F. (2013). Post-Transcriptional Regulation of GORK Channels by Superoxide Anion Contributes Towards Increases in Outward Rectifying K^+^ Currents. New Phytol..

[41] Tran D., Molas M.L., Kadono T., Errakhi R., Briand J., Biligui B., Kawano T., Bouteau F. (2013). A Role for Oxalic Acid Generation in Ozone-Induced Programmed Cell Death. Plant Cell Environ..

[42] Tran D., Rossi M., Biligui B., Kawano T., Mancuso S., Bouteau F. (2013). Ozone-Induced Caspase-Like Activities Are Dependent on Early Ion Channel Regulations and ROS Generation in *Arabidopsis Thaliana* Cells. Plant Signal. Behav..

[43] McCarthy J., Gong X., Nahirney D., Duszyk M., Radomski M. (2011). Polystyrene Nanoparticles Activate Ion Transport in Human Airway Epithelial Cells. Int. J. Nanomed..

[44] Oukarroum A., Bras S., Perreault F., Popovic R. (2012). Inhibitory Effects of Silver Nanoparticles in Two Green Algae, *Chlorella Vulgaris* and *Dunaliella Tertiolecta*. Ecotoxicol. Environ. Safety.

[45] Chen P., Powell B.A., Mortimer M., Ke P.C. (2012). Adaptive Interactions between Zinc Oxide Nanoparticles and *Chlorella* sp. Environ. Sci. Technol..

[46] Atha D.H., Wang H., Petersen E.J., Cleveland D., Holbrook R.D., Jaruga P., Dizdaroglu M., Xing B., Nelson B.C. (2012). Copper Oxide Nanoparticle Mediated DNA Damage in Terrestrial Plant Models. Environ. Sci. Technol..

[47] Slomberg D.L., Schoenfisch M.H. (2012). Silica Nanoparticle Phytotoxicity to *Arabidopsis Thaliana*. Environ. Sci. Technol..

[48] Khodakovskaya M.V., de Silva K., Biris A.S., Dervishi E., Villagarcia H. (2012). Carbon Nanotubes Induce Growth Enhancement of Tobacco Cells. ACS Nano.

[49] Schroeder J.I., Kwak J.M., Allen G.J. (2001). Guard Cell Abscisic Acid Signalling and Engineering of Drought Hardiness in Plants. Nature.

[50] Murashige T., Skoog F. (1962). A Revised Medium for Rapid Growth and Bioassays with Tobacco Tissue Cultures. Physiol. Plant..

[51] Bouizgarne B., El-Maarouf-Bouteau H., Frankart C., Reboutier D., Madiona K., Pennarun A.M., Monestiez M., Trouvier J., Amiar Z., Briand J. (2006). Early Physiological Responses of *Arabidopsis Thaliana* Cells to Fusaric Acid: Toxic and Signaling Effects. New Phytol..

[52] Finkel A.S., Redman S. (1984). Theory and Operation of a Single Microelectrode Voltage Clamp. J. Neurosci. Methods.

[53] Bouteau F., Tran D., Volkov A.G. (2012). Plant response to stress: Microelectrode voltage clamp studies. Plant Electrophysiology.

