# Erratum: Tran et al. Early Cellular Responses Induced by Sedimentary Calcite-Processed Particles in Bright Yellow 2 Tobacco Cultured Cells. *Int. J. Mol. Sci.* **2020**, *21*, 4279

**DOI:** 10.3390/ijms22136863

**Published:** 2021-06-25

**Authors:** Daniel Tran, Tingting Zhao, Delphine Arbelet-Bonnin, Takashi Kadono, Patrice Meimoun, Sylvie Cangémi, Tomonori Kawano, Rafik Errakhi, François Bouteau

**Affiliations:** 1 Agroscope, Institute for Plant Production Systems, 1964 Conthey, Switzerland; 2 Laboratoire Interdisciplinaire des Energies de Demain, Université de Paris, 75013 Paris, France; zhaotingting0325@gmail.com (T.Z.); delphine.bonnin@univ-paris-diderot.fr (D.A.-B.); kadono.takashi@gmail.com (T.K.); patrice.meimoun@upmc.fr (P.M.); sylvie.cangemi@univ-paris-diderot.fr (S.C.); francois.bouteau@univ-paris-diderot.fr (F.B.); 3 Cogitamus Laboratory, 75013 Paris, France; 4 Graduate School of Environmental Engineering, University of Kitakyushu, 1-1 Hibikino, Wakamatsu-ku, Kitakyushu 808-0135, Japan; kawanotom@gmail.com; 5 LINV Kitakyushu Research Center (LINV@Kitakyushu), Kitakyushu 808-0135, Japan; 6 International Photosynthesis Industrialization Research Center, The University of Kitakyushu, Kitakyushu 808-0135, Japan; 7 Paris Interdisciplinary Energy Research Institute (PIERI), Université de Paris, 75013 Paris, France; 8 Eurofins Agriscience Service, Casablanca 20000, Morocco; r.errakhi@gmail.com

The authors would like to remove the scientific consortium ‘Camille Nous’ from the author list and the Author Contributions section in the published paper [1], as suggested by the Editorial Office. To recognize the consortium’s contribution, the authors claimed a double affiliation of two of the authors to the Cogitamus laboratory [2]. The original article has been updated.
