# Supplementary material for: Early Cellular Responses Induced by Sedimentary Calcite-Processed Particles in Bright Yellow 2 Tobacco Cultured Cells
Source: Int J Mol Sci. 2020 Jun 16;21(12):4279. doi: 10.3390/ijms21124279 (PMC7349144; doi:10.3390/ijms21124279)
Supplement: Supplementary file 1 [file ijms-21-04279-s001.pdf]

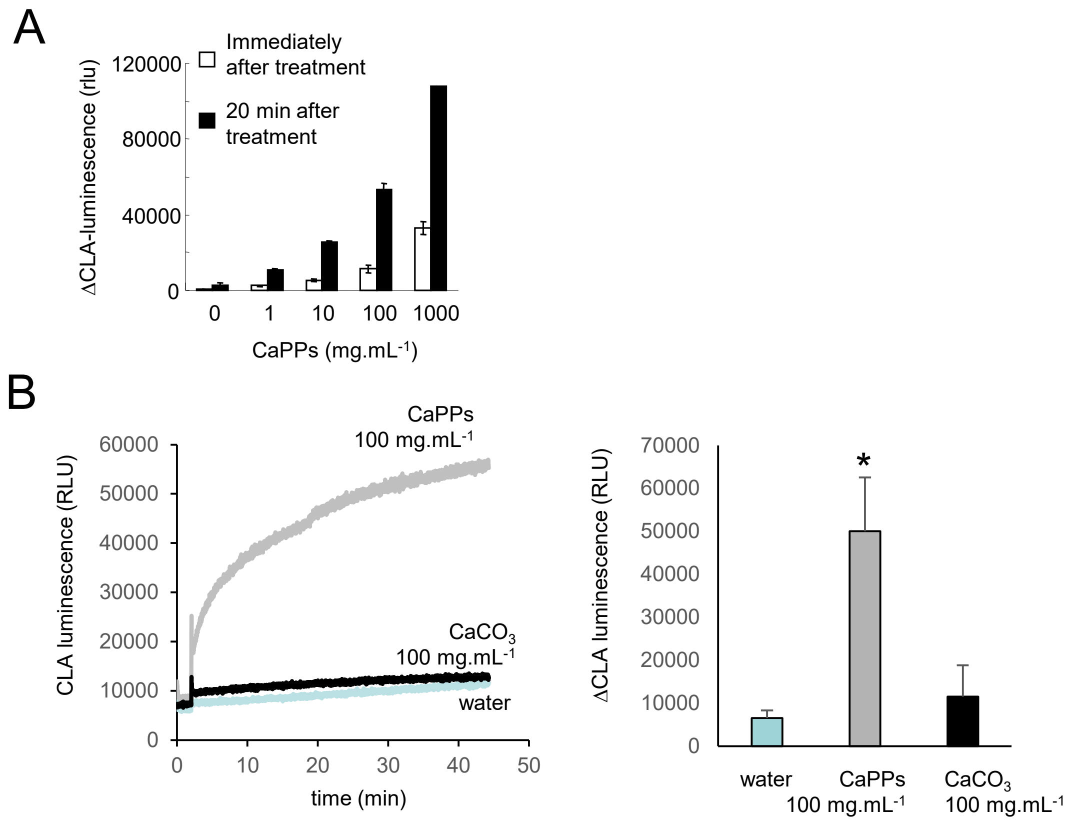

Figure S1. A. Mean values of CaPPs-induced CLA luminescence. **B.** Comparison of CaPPs- and $CaCO_3$-induced ROS generation in free MS medium. Typical time dependent CLA luminescence increase recorded in MS medium after addition of water or, CaPPs or $CaCO_3$ at 100 mg. mL^{-1}. Mean values of CLA chemiluminescence increase 40 min after addition of water, CaPPs or $CaCO_3$. Data correspond to mean values ± SD of at least six independent experiments. * significantly different from the control with water (p-values ≤ 0.05).

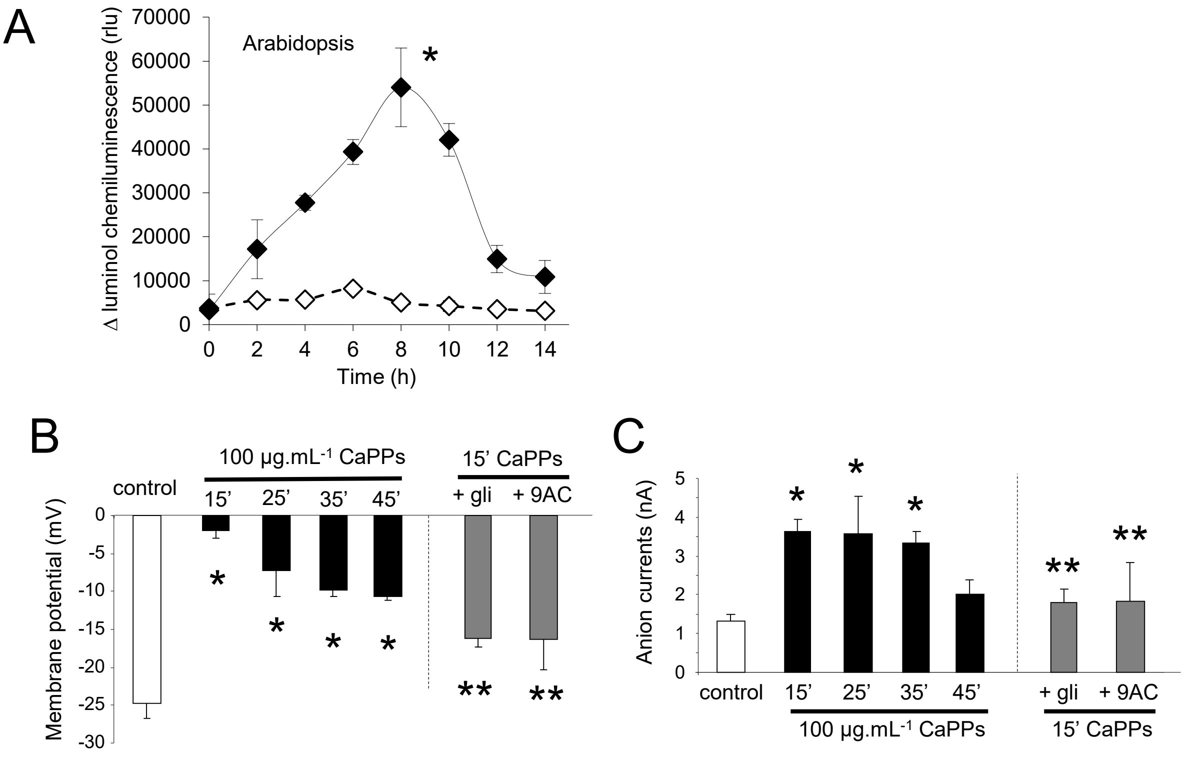

Figure S2 : *Arabidopsis thaliana* L. cultured cells, cell line T87 generated from the ecotype Columbia plant, were grown in Gamborg medium complemented with 20 g.L^{-1} sucrose, 2 mg.L^{-1} 2,4 D, 0.1 mg.L^{-1} kinetin at 22 ± 2 ° C under continuous white light (40 µE.m^{-2}.s^{-1}) with continuous shaking (gyratory shaker at 120 rpm), as previously described (Kadono *et al.* 2010). Cell cultures were sub-cultured weekly using a 1:10 dilution. All experiments were performed at 22 ± 2 ° C using log-phase cells (4 days after sub-culture).
A. Kinetic of biological ROS generation detected with luminol during 7 h after addition of 100 µg.mL^{-1} CaPPs. **B.** Mean values of polarizations for *A. thaliana* cells treated during different times with 100 µg.mL^{-1} CaPPs and mean values of polarizations for *A. thaliana* cells treated 15 minutes with 100 µg.mL^{-1} CaPPs in presence of 200 µM glibenclamide (gli) or 200 µM 9-antharcen carboxylic acid (9AC), two unrelated anion channel inhibitors. **C.** Mean values of anion currents for *A. thaliana* cells treated during different times with 100 µg.mL^{-1} CaPPs and mean values of anion currents for *A. thaliana* cells treated 15 minutes with 100 µg.mL^{-1} CaPPs in presence of 200 µM gli or 200 µM 9AC. Currents were recorded at -200 mV and 1.8 s of voltage clamp. (See Tran *et al.* 2013 for methods related to electrophysiological on *A. thaliana*). Data correspond to mean values ± SD of at least six independent experiments. * significantly different from the control (p-values ≤ 0.05). ** significantly different from the treatment at 15 min (p-values ≤ 0.05).

.

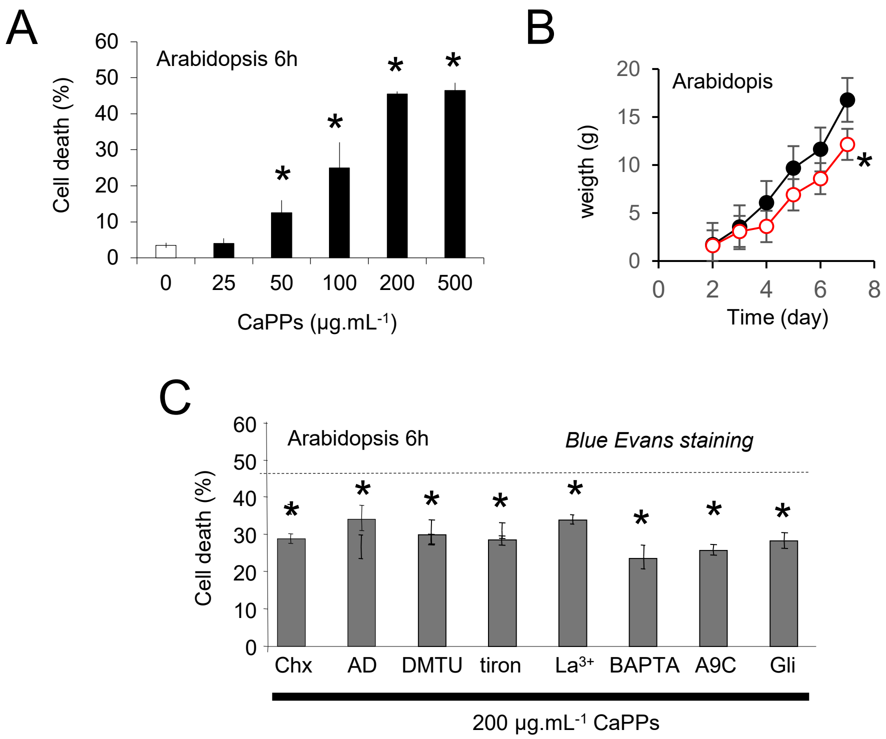

Figure S3 : A. Dose dependent cell death reaching about 50 % of the *Arabidopsis thaliana* cell population was observed after 6 h after treatment with 200 µg. mL^{-1} CaPPs. **B.** Decrease of the culture growth induced by 200 µg. mL-1 CaPPs. Measurements were made on 50 mL of suspension cells. **C.** Decrease of cell death extent by pretreatments with actinomycin D (AD, 20 µg/ml), cycloheximide (Chx, 20 µg/ml), inhibitors of traduction and translation, ROS scavengers Tiron (5mM) and DMTU (100 mM), Ca^{2+} channel blocker La^{3+} (500 µM), Ca^{2+} chelator, BAPTA (3 mM), and anion channel blockers, glibenclamide (gli 200 µM) and 9AC (200 µM). For each pretreatments, cells were incubated for 15 min before CaPPs treatment. The dashed line correspond to the cell death extent induced by 200 µg.mL^{-1} CaPPs. * significantly different from the control (p-values ≤ 0.05).

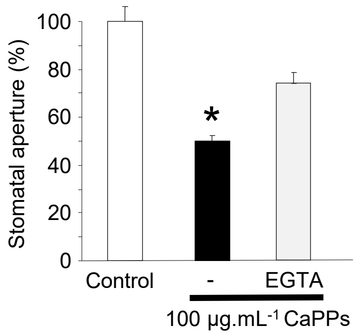

Figure S4: Applications of 100 µg.mL^{-1} CaPPs reduce the stomatal aperture of *A. thaliana* leaves. In presence of 3 mM EGTA, the CaPPs-induced stomatal closure was reduced. Epidermal strips were carefully prepared from abaxial epidermis then placed cuticule side-down on microscope slides covered with medical adhesive and immediately floated in 10 mM MES pH 6.1, 50 mM KCl, 1 mM $CaCl_2$ (opening buffer) under white light (40 µmol photons m^{-2} s^{-1}) for 3 h before treatment. Means of 100 measurements of individual stomata, with standard errors. * significantly different from the control (p-values ≤ 0.05).
